# Stroke nurse navigator utilization reduces unplanned 30-day readmission in stroke patients treated with thrombolysis

**DOI:** 10.3389/fneur.2023.1205487

**Published:** 2023-06-15

**Authors:** Adalia H. Jun-O'Connell, Eliza Grigoriciuc, Akanksha Gulati, Brian Silver, Kimiyoshi J. Kobayashi, Majaz Moonis, Nils Henninger

**Affiliations:** ^1^Departments of Neurology, University of Massachusetts Chan Medical School, Worcester, MA, United States; ^2^Departments of Internal Medicine, University of Massachusetts Chan Medical School, Worcester, MA, United States; ^3^Departments of Psychiatry, University of Massachusetts Chan Medical School, Worcester, MA, United States

**Keywords:** stroke, TPA, stroke nurse navigator, 30-day readmission, quality improvement

## Abstract

**Background:**

Unplanned 30-day hospital readmissions following a stroke is a serious quality and safety issue in the United States. The transition period between the hospital discharge and ambulatory follow-up is viewed as a vulnerable period in which medication errors and loss of follow-up plans can potentially occur. We sought to determine whether unplanned 30-day readmission in stroke patients treated with thrombolysis can be reduced with the utilization of a stroke nurse navigator team during the transition period.

**Methods:**

We included 447 consecutive stroke patients treated with thrombolysis from an institutional stroke registry between January 2018 and December 2021. The control group consisted of 287 patients before the stroke nurse navigator team implementation between January 2018 and August 2020. The intervention group consisted of 160 patients after the implementation between September 2020 and December 2021. The stroke nurse navigator interventions included medication reviews, hospitalization course review, stroke education, and review of outpatient follow-ups within 3 days following the hospital discharge.

**Results:**

Overall, baseline patient characteristics (age, gender, index admission NIHSS, and pre-admission mRS), stroke risk factors, medication usage, and length of hospital stay were similar in control vs. intervention groups (*P* > 0.05). Differences included higher mechanical thrombectomy utilization (35.6 vs. 24.7%, *P* = 0.016), lower pre-admission oral anticoagulant use (1.3 vs. 5.6%, *P* = 0.025), and less frequent history of stroke/TIA (14.4 vs. 27.5%, *P* = 0.001) in the implementation group. Based on an unadjusted Kaplan–Meier analysis, 30-day unplanned readmission rates were lower during the implementation period (log-rank *P* = 0.029). After adjustment for pertinent confounders including age, gender, pre-admission mRS, oral anticoagulant use, and COVID-19 diagnosis, the nurse navigator implementation remained independently associated with lower hazards of unplanned 30-day readmission (adjusted HR 0.48, 95% CI 0.23–0.99, *P* = 0.046).

**Conclusion:**

The utilization of a stroke nurse navigator team reduced unplanned 30-day readmissions in stroke patients treated with thrombolysis. Further studies are warranted to determine the extent of the results of stroke patients not treated with thrombolysis and to better understand the relationship between resource utilization during the transition period from discharge and quality outcomes in stroke.

## Introduction

Unplanned hospital readmission following a stroke is a costly and common problem in the United States (1). Reported unplanned readmission rates after a stroke have been reported to be as high as 12–21% within 30 days, reaching up to 55% within 1 year (2–6). The use of intravenous tissue plasminogen activator (tPA) improves long-term outcomes (7–12). Moreover, it has shown 11 to 23% lower odds of 30-day unplanned readmission (6), indicating that there is a significant subset of tPA-treated patients that is at risk for unplanned 30-day readmission. Therefore, quality improvement measures are needed to further reduce readmission risk. Specifically, the transition period between the hospital discharge and subsequent ambulatory follow-up following an ischemic stroke is a critical period during which medication errors, failed hand-offs, inadequate post-discharge support, and loss of follow-up can occur, which may increase the risk for complications and unplanned readmission (13–15). Thus, there is a critical need to optimize systems of care in clinical practice to improve post-stroke outcomes (16, 17).

One strategy to improve patient care during the transition period may include post-discharge phone calls, which have been shown to reduce unplanned hospital readmission rates within 6 months in a pragmatic, randomized control trial (18). However, little is known whether such an intervention reduces readmission rates in patients treated with thrombolysis and whether this translates to improved long-term outcomes (16).

To address this issue, we sought to determine whether the utilization of a stroke nurse navigator team reduces 30-day stroke unplanned readmission of tPA-treated patients during the transition period. Comparisons between tPA-treated patients vs. non-tPA-treated patients were made as we recognize the concern that there remains a significant subset of tPA-treated patients that is at risk for unplanned 30-day readmission. The tPA-treated patients offer an additional advantage in the study, considering that all patients with acute ischemic strokes undergo a similar work-up and treatment paradigm in the acute setting due to the standardization of stroke care. A secondary objective was to determine whether this intervention was associated with a reduced risk of major adverse cardiovascular events (MACE), functional deficit severity defined by the modified Rankin scale (mRS), and neurological status defined by the National Institutes of Health Stroke Scale (NIHSS) at 90-days from discharge.

## Methods

### Study cohort

We retrospectively analyzed prospectively accrued adult patients (age 18 years and older) who were admitted to our academic tertiary care center for an acute ischemic stroke between January 2018 and December 2021. Electronic medical records and relevant ICD codes were used to identify the principal diagnosis of stroke. Patients who were determined to have had a planned 30-day readmission as identified by our hospital readmission committee were excluded from this search. We excluded patients who did not receive intravenous thrombolysis, died during the index admission, were discharged to hospice, had a hospital length of stay exceeding 30 days, or were lost to follow-up (19). The Institutional Review Board (IRB) approved the study, and the Health Insurance Portability and Accountability Act (HIPPA) waiver of informed consent was granted. We prepared our manuscript according to the Strengthening the Reporting of Observational Studies in Epidemiology guidelines (http://www.strobe-statement.org).

All diagnoses were first established by the treating board-certified neurologist and confirmed by abstracting physicians (EG and AG). Conflicting diagnoses were resolved by consensus after adjudication by board-certified vascular neurologists (AJ-O and NH).

### Intervention

We compared patients treated before nurse navigator implementation (between January 2018 and August 2020) with those treated after nurse navigator implementation (between September 2020 and December 2021). The stroke nurse navigator team consisted of two trained nurses (RN) experienced in stroke care. After the nurse navigator implementation, each patient received a standardized follow-up transition plan as follows. On the day of the discharge, introduction to the transition process, ambulatory follow-up appointments, and ambulatory testing were confirmed. Between days 3 and 7 after discharge, nurses conducted phone interviews with the patients and/or their health caregiver to review discharge summaries, verify medications, confirm the follow-up plans for outpatient-based testing and appointments, and address patient satisfaction and any outstanding questions. The flow process was such that each stroke nurse navigator had access to the inpatient stroke admission team list, and they attended daily huddles twice a week on Monday and Wednesday. On the day of the discharge, the inpatient team notifies the stroke nurse navigator for disposition follow-up plans. To prevent potential missed errors, the stroke nurse navigator reviewed the inpatient stroke team census each morning to identify potential discharge candidates and verified the discharge plan with the stroke team *via* electronic communication. To determine whether our goal to have the nurse navigator call patients within 3–7 days was met, we spot-checked every other patient (*n* = 81). Among these, the median time to patient call was 3 days (interquartile range 2–7 days) after discharge. Any issues that the nurse navigator could not resolve were escalated to the discharging physicians and neurology quality officer (AJ-O) for resolution.

### Data collection

Patient age, gender, insurance information, total admission cost in dollars, index admission length of stay (LOS), co-morbidities, pre-admission medications, admission National Institutes of Health Stroke Scale (NIHSS), admission modified Rankin Score (mRS), and discharge status were collected for all patients by review of the medical records through the electronic medical record system. In addition, we assessed COVID-19 status (confirmed infection within 30 days of the index admission) in all patients that were admitted between 2020 and 2021.

### Definitions

The transition of care was defined as the movement of a patient from the admitted hospital to another healthcare setting (20). The index admission was defined as the admission of the starting point for studying repeat hospital visits (21). The 30-day unplanned readmission was defined as a subsequent unplanned admission, occurring within 30 days of the discharge date from the index admission (22). For dyslipidemia, two definitions were used: LDL higher than 100 based upon AHA/ASA ischemic stroke guideline (23) and LDL higher than 189 based upon ACC/AHA guideline (24).

### Study outcomes

The primary outcome of interest was the 30-day unplanned readmission. Secondary outcomes of interest were the rate of MACE, including death within 90 days from discharge as well as the 90-day mRS and NIHSS. Medical records were independently reviewed by two investigators to confirm the qualifying index diagnosis and the outcomes of interest.

### Statistical analyses

Data are reported as median (interquartile range) unless otherwise stated. Univariate comparisons were performed using the χ^2^ test, Fisher's exact test, and Mann-Whitney U-test as appropriate. A two-sided *p*-value of < 0.05 was considered to be statistically significant in all analyses. The Kaplan–Meier analysis, log-rank test, and multivariable Cox regression analysis (with backward elimination) were used to determine whether stroke nurse navigator implementation was associated with a reduction of the 30-day unplanned readmission rate. We calculated adjusted hazard ratios (aHR) with corresponding 95% confidence intervals (CI). Models were adjusted for age, gender, admission NIHSS, pre-admission mRS, treatment with mechanical thrombectomy, oral anticoagulant use, history of stroke/TIA, COVID-19 diagnosis within the 30 days of index admission, and total cost of the index hospitalization. All statistical analyses were performed using IBM SPSS Statistics version 28.0.1 (IBM, Armonk, NY).

## Results

**Figure 1:**
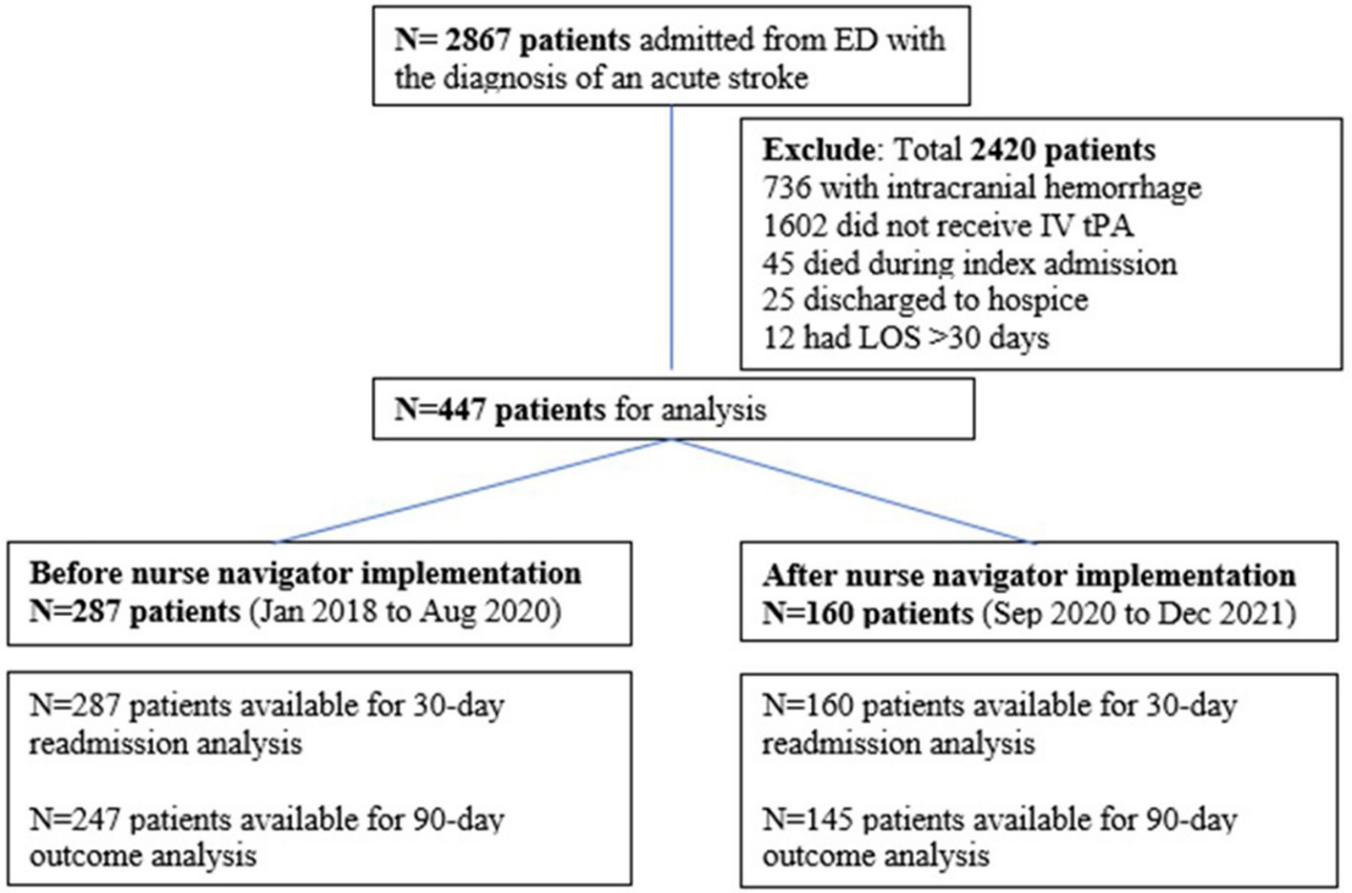
Patient flowchart.

The study flowchart is depicted in Figure 1. We included 447 consecutive stroke patients treated with thrombolysis (*n* = 287 before and *n* = 160 after the implementation of stroke nurse navigator transition care implementation).

Table 1 shows the baseline characteristics of the studied population as stratified by a stroke nurse navigator transition care implementation status. Overall, patient characteristics were similar between groups except for higher mechanical thrombectomy utilization (*P* = 0.016), lower pre-admission oral anticoagulant use (*P* = 0.025), less frequent history of stroke/TIA (*P* = 0.001), more frequent COVID-19 diagnosis (*P* = 0.001), and higher total cost of hospitalization (*P* < 0.001) in the implementation vs. the control group.

**Table 1:** Patient characteristics stratified by absence vs. presence of stroke nurse navigator implementation in ischemic strokes that received alteplase.

**Characteristics**	**Before implementation (*n =* 287)**	**After implementation (*n =* 160)**	***P*-value**
Age [Years; Median (IQR)]	**71 (58–80)**	**69 (59–78)**	0.54
Gender			0.37
Female	128 (44.6%)	79 (49.4%)	
Male	159 (55.4%)	81 (50.6%)	
Total cost of hospitalization ($), median (IQR)	$22,989 ($17,192–$34,571)	$27,096 ($21,896–$53,208)	< 0.001
Index admission NIHSS, median (IQR)	6 (3–12)	7 (3–14)	0.53
Pre-admission mRS, median (IQR)	0 (0–1)	0 (0–1)	0.2
Length of stay, median (IQR)	3 (2–6)	4 (2–6)	0.54
Mechanical thrombectomy	71 (24.7%)	57 (35.6%)	0.02
Dyslipidemia			
LDL > 100	99 (34.9%)	61 (38.1%)	0.54
LDL > 189	5 (1.8%)	3 (1.9%)	1
History of prior stroke or TIA	79 (27.5%)	23 (14.4%)	0
Hypertension	212 (73.9%)	114 (71.3%)	0.58
Diabetes mellitus	72 (25.1%)	43 (26.9%)	0.74
Atrial fibrillation	85 (29.6%)	40 (25%)	0.32
Coronary artery disease	64 (22.3%)	28 (17.5%)	0.27
CHF	43 (15%)	17 (10.6%)	0.25
Peripheral arterial disease	77 (26.8%)	40 (25%)	0.74
Tobacco use hx	141 (49.1%)	70 (43.8%)	0.28
Statin use	129 (44.9%)	76 (47.5%)	0.62
Anti-hypertensives	191 (66.6%)	101(63.1%)	0.47
Anti-diabetic med	56 (19.5%)	25 (15.6%)	0.37
Antiplatelet use	123 (42.9%)	59 (36.9%)	0.23
Oral anticoagulant	16 (5.6%)	2 (1.3%)	0.03
Insurance			0.5
Medicare	165 (57.5%)	89 (55.6%)	
Medicaid	24 (8.4%)	20 (12.5%)	
Commercial	89 (31.0%)	50 (31.3%)	
Military	3 (1.0%)	0 (0%)	
Others	5 (1.7%)	1 (0.6%)	
Uninsured	1 (0.3%)	0 (0%)	
Discharge status			0.56
Home	147 (51.2%)	77 (48.1%)	
Facility	140 (48.8%)	83 (51.9%)	
COVID-19 diagnosis within 30 days of index admission	1 (0.4%)	8 (5.4%)	0.001

Data are median (IQR) or *n* (%). IQR, Interquartile Range; NIHSS, National Institutes of Health Stroke Scale; mRS, Modified Rankin Score; TIA, Transient ischemic attack; Facilities (acute rehabilitation center, short-term or long-term nursing facility).

### Reasons for 30-day unplanned readmission in ischemic stroke

Among the 447 stroke patients, 50 (11.2%) had 30-day unplanned readmission. In total, 16 (32%) readmissions were due to neurological causes (such as seizure or recurrent stroke), and 34 (68%) readmissions were due to medical issues. In the subgroup of readmissions due to medical issues, 12 (24%) were due to infections.

### Association between stroke nurse navigator implementation and 30-day unplanned readmission risk

**Figure 2:**
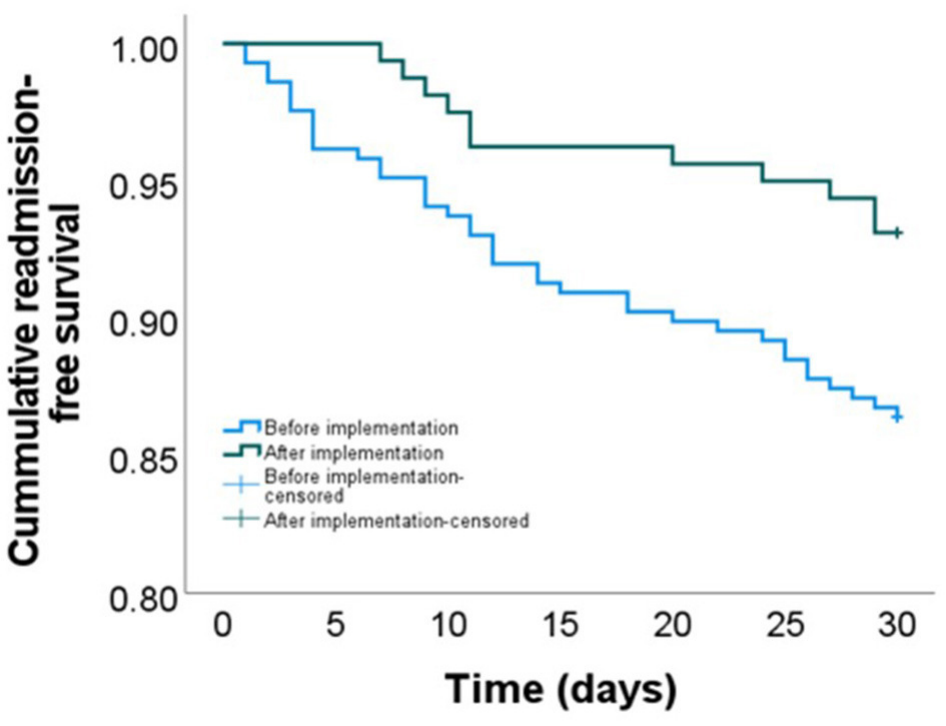
Cumulative unpreventable 30-day readmission-free survival stratified by a stroke nurse navigator implementation period. Log-rank *P* = 0.029; adjusted HR 0.48, 95% CI (0.23–0.99).

Based on an unadjusted analysis, the 30-day readmission rate was significantly higher prior to stroke nurse navigator implementation as compared to post-implementation (13.6% vs. 6.9%, *P* = 0.041) with the Kaplan–Meier analysis indicating continued separation of the readmission rates throughout the 30-day transition period (log-rank *P* = 0.029, Figure 2). The implementation of the nurse navigator transition care remained independently associated with a lower adjusted hazard rate (aHR) of unplanned 30-day readmission based on a multivariable Cox regression analysis (aHR 0.48, 95% CI 0.23–0.99, *P* = 0.046, Table 2). Overall, patients with the stroke nurse implementation had a 67.6% reduced probability (defined as 1-[aHR/(1+aHR)]) of 30-day unpreventable stroke readmission compared to patients without the implementation.

**Table 2:** Variables associated with 30-day unplanned readmission on multivariable Cox regression.

**Study variable**	**Hazard ratio (95% CI)**	***p*-value**
Nurse navigator implemented	0.48 (0.23–0.99)	0.046
NIHSS	1.04 (1–1.08)	0.052
Thrombectomy	0.47 (0.22–1.01)	0.053
Total admission cost (per $10,000)	1.12 (1.02–1.23)	0.021
History of stroke and or TIA	1.93 (1.07–3.45)	0.028

CI, confidence interval. Variables not retained in the final step of the model included age, gender, history of stroke/TIA, oral anticoagulant use, pre-admission mRS, and COVID-19 infection within 30 days.

### 90-day MACE

Table 3 summarizes the univariate comparison of 90-day outcomes between groups. Overall, we found no significant differences in the unadjusted 90-day mortality, MACE, 90-day NIHSS, and 90-day mRS (*P* > 0.05, each).

**Table 3:** Outcome events.

**Characteristics**	**Before implementation (*n =* 287)**	**After implementation (*n =* 160)**	***P*- value**
30-day Readmission	39 (13.6%)	11 (6.9%)	0.041
90-day Outcome			0.313
Death	16 (5.6%)	11 (6.9%)	
Cardiovascular event	31 (10.8%)	11 (6.9%)	

## Discussion

Despite improvements in stroke prevention and acute stroke care processes, many patients and their caregivers face significant gaps in post-stroke care during the post-stroke recovery period (16). A recent systemic review and meta-analysis of qualitative studies focusing on hospital-to-home transition care in stroke showed the importance of patient and caregiver engagement in discharge preparation, along with the need for the implementation of post-discharge support to help stroke patients adjust to post-stroke rehabilitation and the need for integrated transitional support for post-stroke adjustment (25). However, the potential impact of stroke nurse navigator implementation on unplanned readmissions in patients treated with thrombolysis is unknown.

We now show that in the studied cohort, the utilization of a stroke nurse navigator for transition care was independently associated with a reduced rate of unplanned 30-day readmission in ischemic stroke patients treated with thrombolysis. This is an important finding as the Centers for Medicare and Medicaid Services (CMS) defined 30-day unplanned readmission as an indicator of poor hospital care, connecting to hospital penalties and payment determination (2, 26). The utilization of stroke nurse navigator teams for the transition period may thus represent an effective tool to improve stroke care by both engaging patients and caregivers to improve support as well as reduce unplanned readmission risk. This is important as stroke nurse navigators during the transition period are also known to improve self-efficacy, quality of life, and stroke-relevant knowledge, as well as reduce caregiver burnout (27). Further studies aimed at identifying patients at high risk for unplanned readmission have the potential to address precipitating factors and create an opportunity to recognize and mitigate issues surrounding patient care during the transition period (1).

Our study also showed a 67.6% reduced probability of 30-day unplanned readmission with transition care utilization. Although the previous Transition Coaching for Stroke (TRACS) trial showed a potential reduction of 30-day unplanned readmissions following stroke by 48% with transition care, the study called for further replicable studies due to its size of 510 patients in a single center setting, and the study intervention focused only on patients that were discharged home (17). Contrary to TRACS, our study also included patients that were discharged to facilities.

Previous studies showed that unplanned 30-day readmission after stroke is associated with increased mortality and significant societal financial burden, costing up to 17 billion dollars in the United States alone (2). Therefore, we sought to determine whether stroke nurse navigator utilization could improve the risk of MACE including mortality as well as functional outcomes by 90 days after stroke. We found no significant difference in these outcomes between studied cohorts. This observation is similar to the COMPASS (Comprehensive Post-Acute Stroke Services) trial, which failed to show that post-acute stroke transitional care services had a significant effect on the 90-day functional outcome as assessed by the Stroke Impact Scale (primary outcome) as well as the mRS and mortality outcome (secondary outcomes). This may have been in part related to the pragmatic trial design with incomplete case ascertainment (16). Nevertheless, our results should be interpreted cautiously as subjects were not randomly assigned to the intervention, and comparison groups were not measured during concurrent time periods introducing the possibility of unmeasured confounding. Thus, further prospective studies in larger cohorts are required to determine the possible beneficial effects of stroke nurse navigator implementation on long-term outcomes.

The CMS created the Bundled Payments of Care Improvement (BPCI) initiative, which created incentives for cost and quality care (28). Although there is limited data, the impact of stroke bundle programs is being studied on patient outcomes, and stroke nurse navigators are starting to be recognized to provide support for BPCI initiatives (28, 29). From a patient perspective, there is a demand and need for having a dedicated care coordinator or point of contact during the transition period to guide through expectations of post-stroke adjustment and transition (30–33).

Further future studies are needed to investigate the cost-effectiveness of stroke nurse navigators in stroke BPCI initiative, quality improvements, and overall patient satisfaction. Patients that are specifically discharged home with home healthcare have been shown to have a higher 30-day unplanned readmission rate and significantly lower Medicare payment reimbursements for overall care within the first 60 days after index admission (34). Thus, the optimization and standardization of transition care services also need to be further considered in the future for better patient support (1). Future studies are warranted to further understand the association between stroke nurse navigators, patient outcomes, and barriers to improvement in care. Specifically, it will be important to study the associative relationship between patient outcomes and medication compliance, access to ambulatory services and treatments, follow-up appointments, and issues surrounding patient-nurse navigator communications. It is also not known if families used other outpatient services more after implementation. One may hypothesize that patients were less willing to bring loved ones to the hospital due to the pandemic or other concerns and utilized outpatient services more. Future studies are needed to further address the question of potential increased usage of resource utilization after stroke nurse navigator implementation.

There are limitations to this study. As discussed, comparison groups were not measured during concurrent time periods, which may have introduced the possibility that unmeasured factors may have played a role in the differences seen. Furthermore, the sample sizes and time frame of the pre-vs. post-implementation phase differed. While this approach may have introduced additional bias, it allowed us to increase the overall sample size and, thus, the power of our analyses. Second, the study population was obtained from a single tertiary care center limiting the generalizability of our findings. Nevertheless, the observed unplanned 30-day readmission rate was 13.6% prior to stroke nurse navigator implementation, which is in line with previously reported rates of 12 to 21% (1), suggesting that our results likely translate to other hospital settings. Furthermore, although we adjusted our analyses for COVID-19 status, we cannot exclude the possibility that our results are biased by unmeasured factors related to the COVID-19 pandemic. Finally, there were differences in the total cost of care in the intervention vs. non-intervention despite the similar length of stay. The difference may be explained by the greater number of thrombectomies in the intervention, which could potentially increase the hospital cost. This could confound our results and therefore should be interpreted with caution. However, it also attests to the operational strength, in which the volume of thrombectomy cases did not decrease during the COVID-19 pandemic.

## Conclusion

The utilization of a stroke nurse navigator team was associated with reduced unplanned 30-day readmissions in stroke patients treated with thrombolysis. Further prospective studies are warranted to determine the extent of the results of stroke patients not treated with thrombolysis and to better understand the relationship between resource utilization during the transition period from discharge and quality outcomes in stroke.

## Data availability statement

The raw data supporting the conclusions of this article will be made available on reasonable request. Access requests should be directed to the corresponding author(s).

## Ethics statement

The studies involving human participants were reviewed and approved by IRB at UMass Chan Medical School. Written informed consent for participation was not required for this study in accordance with the national legislation and the institutional requirements.

## Author contributions

AJ-O'C: study concept and design, data acquisition, interpretation of data, drafting of the manuscript, and critical revision of the manuscript for important intellectual content. EG: data acquisition, interpretation of data, and critical revision of the manuscript for important intellectual content. AG: data acquisition and critical revision of the manuscript for important intellectual content. BS: study concept, interpretation of data, and critical revision of the manuscript for important intellectual content. KK and MM: critical revision of the manuscript for important intellectual content. NH: study concept and design, interpretation of data, drafting of the manuscript, data analysis, and critical revision of the manuscript for important intellectual content. All authors contributed to the article and approved the submitted version.
